# Case Report: Intravascular Ultrasound-guided Intervention for Anastomosis Stenosis of the Left Main Coronary Artery Post-Cabrol Technique

**DOI:** 10.3389/fcvm.2022.778815

**Published:** 2022-03-02

**Authors:** Seok Oh, Ju Han Kim, Dae Young Hyun, Kyung Hoon Cho, Min Chul Kim, Doo Sun Sim, Young Joon Hong, Youngkeun Ahn, Myung Ho Jeong, Yochun Jung

**Affiliations:** ^1^Department of Cardiology, Chonnam National University Hospital, Gwangju, South Korea; ^2^Department of Cardiology, Chonnam National University Medical School, Gwangju, South Korea; ^3^Department of Thoracic and Cardiovascular Surgery, Chonnam National University Hospital, Gwangju, South Korea

**Keywords:** intravascular ultrasound, percutaneous coronary intervention, aortocoronary graft, acute myocardial infarction, Behçet's disease

## Abstract

**Introduction:**

Some cases of percutaneous coronary intervention (PCI) for the anastomotic site between the Cabrol-type conduit and the left main coronary artery (LMCA) have been reported. Nevertheless, the combination of PCI with a detailed description of lesion appearance using virtual histology-intravascular ultrasound (VH-IVUS) has never been reported. In this study, we present a case of acute myocardial infarction that was successfully treated with intravascular ultrasound (IVUS)-guided PCI for focal stenosis at the anastomotic site, and the plaque composition was studied in detail.

**Case Presentation:**

A 35-year-old Korean male with Behçet's disease was diagnosed with acute myocardial infarction. He had previously undergone three cardiothoracic surgeries including two aortic replacements, followed by modified Bentall operation with a Cabrol-type aortocoronary anastomosis. Coronary angiogram (CAG) showed focal critical stenosis at the anastomosis site between the conduit and the LMCA, and VH-IVUS showed fibrotic plaque with mainly fibrous tissue but without a confluent necrotic core. PCI was performed using a drug-eluting stent (4.5 × 12 mm, Synergy^TM^, Boston Scientific, Marlborough, MA, USA). Since a repeat CAG and IVUS post-surgery showed an under-expanded stent strut, post-dilation ballooning was additionally performed. Subsequently, the repeat IVUS revealed wellapposed and optimized deployment of the drug-eluting stent with full lesion coverage. Final CAG showed optimal angiographic results. After successful PCI, the patient's anginal symptoms improved dramatically, and he was successfully discharged from our hospital.

**Conclusion:**

This study presents an IVUS-guided PCI case for an anastomotic site between the conduit and the LMCA. It is the first to investigate the characteristics of this lesion through VH-IVUS, which demonstrated the presence of fibrous plaques at the anastomotic site. IVUS radiofrequency data allow for a detailed assessment of plaque composition and provide new insights into the histopathological nature of stenotic lesions at the anastomotic site, especially in patients with chronic inflammatory diseases like Behçet's disease.

## Introduction

Several surgical techniques have been introduced for re-implantation of the native coronary arteries after aortic root replacement. Bentall and De Bono introduced a novel method of direct re-implantation of epicardial coronary arteries into the aortic graft in 1968 (1). However, this procedure has several limitations owing to postoperative complications including anastomotic bleeding or pseudoaneurysm formation. The Cabrol technique, introduced in 1981, includes interposition of an 8- to 10-mm-diameter Dacron graft between the aortic root and the native coronary artery (2). Because the interposed graft attenuates tension at the coronary–aorta anastomosis, the Cabrol technique is beneficial when a scar from any previous surgery inhibits coronary mobilization.

Whereas the Bentall operation has a very low incidence of coronary stenosis, there have been several reports of graft-coronary anastomosis in the Cabrol and Cabrol-related surgical techniques (3–6). In many cases, this complication is mainly treated through percutaneous coronary intervention (PCI). We found 11 reports about PCI of Cabrol conduit–left main coronary artery (LMCA) anastomosis. However, thus far, cases involving the combination of PCI and virtual histology-intravascular ultrasound (VH-IVUS), which provides a detailed description of lesion appearance, have never been reported. Herein, we describe an unusual case of acute myocardial infarction (AMI) that was successfully treated with IVUS-guided PCI for focal stenosis at the anastomotic site, with a detailed assessment of plaque composition.

## Case Presentation

**Figure 1:**
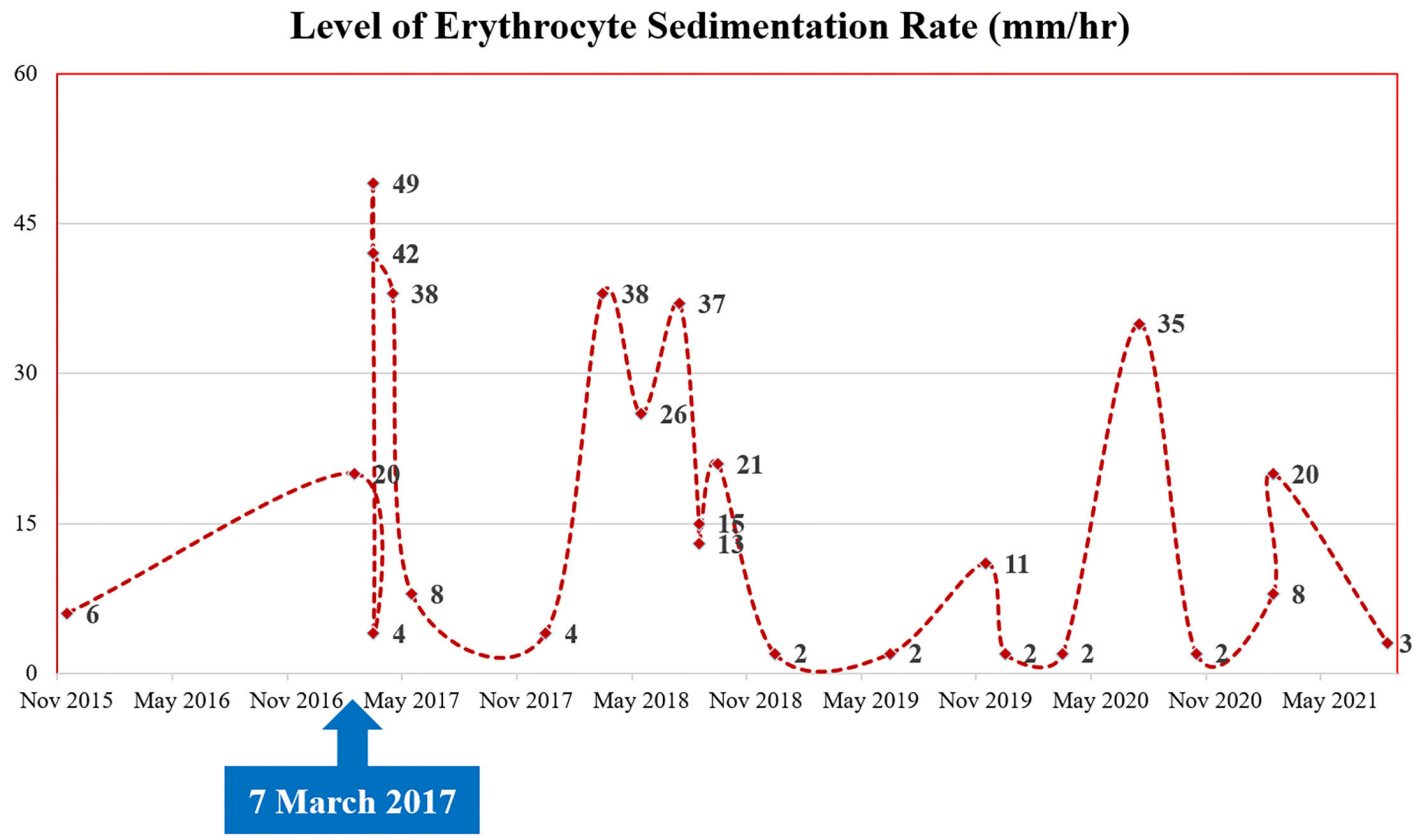
Patient's clinical course: the change of inflammatory markers (levels of erythrocyte sedimentation rate).

A 35-year-old Korean man visited our hospital with a chief complaint of chest pain (Canadian Cardiovascular Society Grade II). The patient had Behçet's disease and aortic regurgitation, which is one of its cardiovascular manifestations. For Behçet's disease, the patient had received anti-inflammatory medications–sulfasalazine (1,000 mg/day); prednisolone (5 mg/day); and colchicine (0.6 mg/day) with high adherence. In the outpatient clinic, laboratory blood parameters including inflammatory markers (erythrocyte sedimentation rate) were routinely checked, which is illustrated in Figure 1. He underwent three cardiothoracic surgeries: aortic valve (AoV) replacement (in 2006) with a 21-mm St. Jude Medical AoV prosthesis (St. Jude Medical, Inc., St. Paul, MN, USA), aortic root replacement (in 2007) with an Edwards Prima stentless porcine prosthesis (Edwards Lifesciences Corp., Irvine, CA, USA) for AoV detachment, and the modified Bentall procedure (on 7 March 2017, ~4 years ago from the time of presentation) for prosthetic valve failure and moderate aortic regurgitation. During the last surgery, a Cabrol-type aortocoronary anastomosis was performed. Because his anginal symptoms gradually deteriorated, the patient visited our cardiovascular center for diagnosis and management.

**Figure 2:**
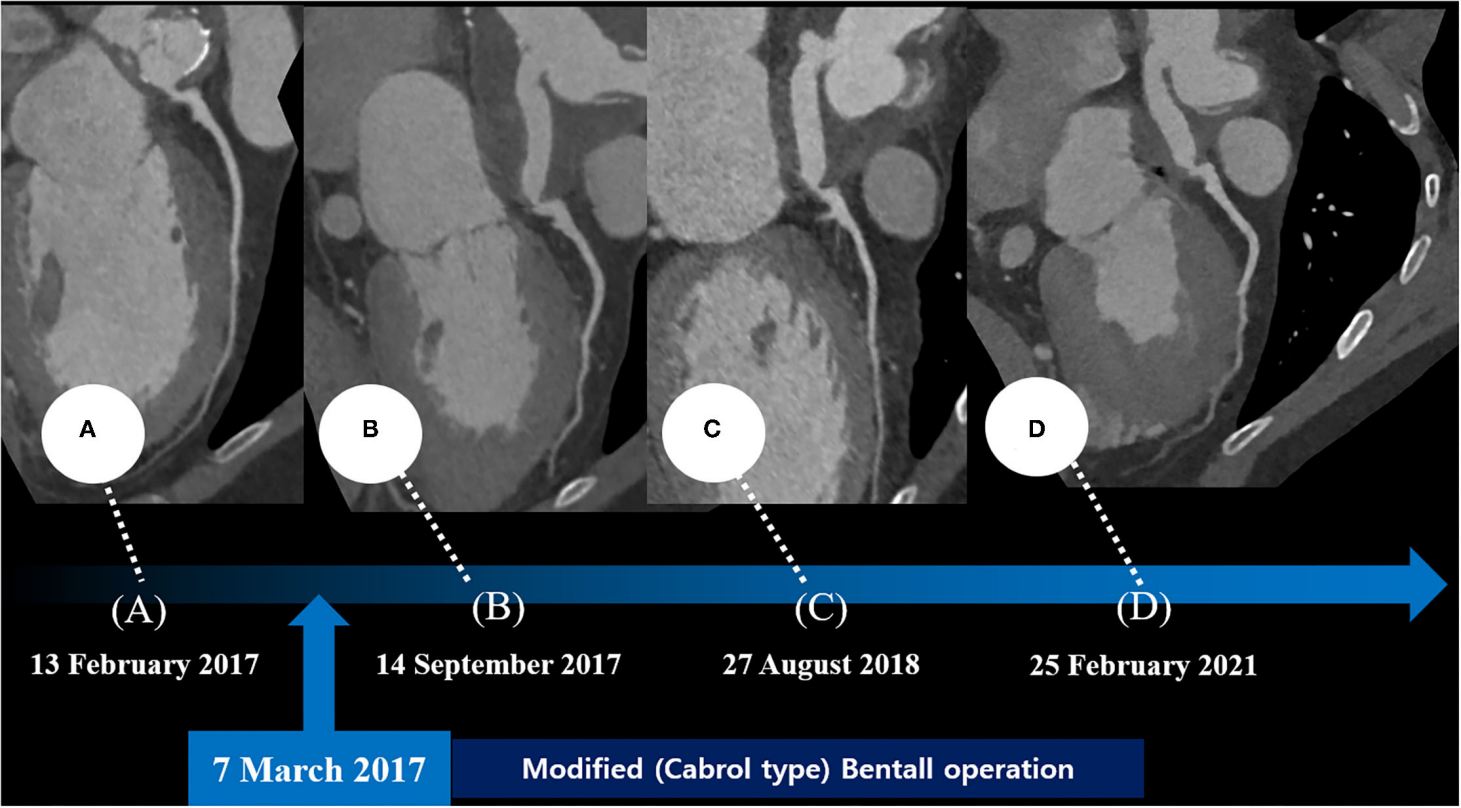
Series of coronary computed tomography angiographies at the preoperative period **(A)** and the postoperative period **(B–D)**.

**Figure 3:**
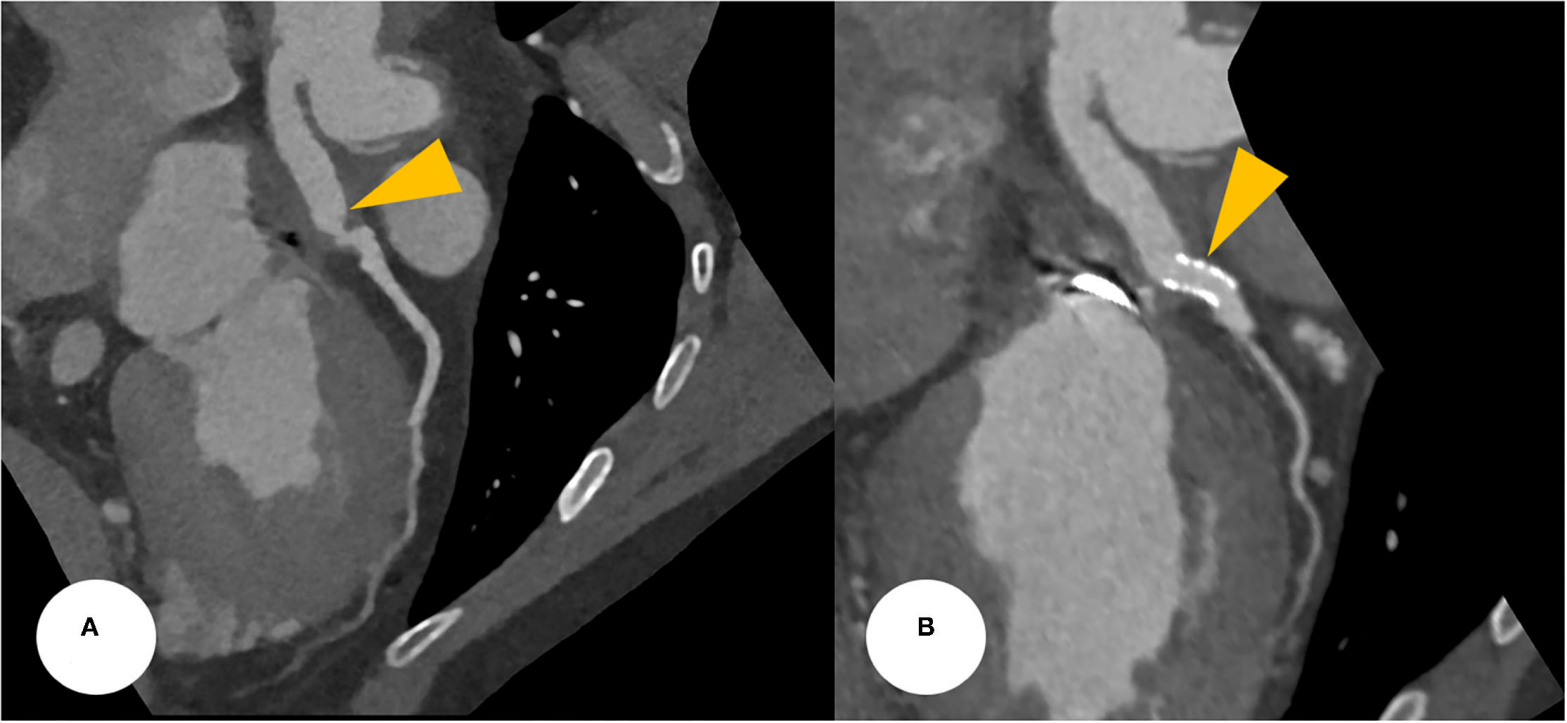
**(A)** CCTA at the postoperative period found a focally narrowed and kinked lesion at the anastomotic site between the Cabrol-type graft and LMCA (a yellowish arrowhead). **(B)** After a successful PCI, CCTA was evaluated, revealing a stent deployment state between the conduit and LMCA with good patency (yellowish arrowheads). CCTA, coronary computed tomography angiography; LMCA, left main coronary artery; PCI, percutaneous coronary intervention.

His vital signs were as follows: temperature, 36.3°C; heart rate, 75 beats/min; respiratory rate, 20 breaths/min; and blood pressure, 110/80 mmHg. A 12-lead electrocardiogram showed normal sinus rhythm with a right bundle branch block. However, the electrocardiogram also revealed ST-segment elevation in aVR and V1, with ST-segment depression in lead I, II, and aVL and precordial leads V4–6, suggesting LMCA occlusion (Supplementary Figure 1). Chest radiography showed mild cardiomegaly and definite evidence of prior median sternotomy and valvular replacement (Supplementary Figure 2). Laboratory tests showed elevated levels of troponin I (0.887 ng/mL; reference: 0–0.050 ng/mL) and pro-brain natriuretic peptide (1,549 pg/mL; reference: 0–300 pg/mL). The patient was administered warfarin, therefore, the prothrombin time–international normalized ratio was estimated to be 1.90. A two-dimensional transthoracic echocardiogram revealed a well-functioning AoV with akinetic movement at the anterior and anteroseptal parts of the myocardium, and a left ventricular ejection fraction of 36.3%. We reviewed the findings of previous coronary computed tomography angiography to obtain detailed anatomical information about the focal stenosis at the anastomotic site between the LMCA and the aortocoronary graft (Figures 2, 3A). Since the patient was diagnosed with non-ST-segment elevation acute coronary syndrome, PCI was performed.

**Figure 4:**
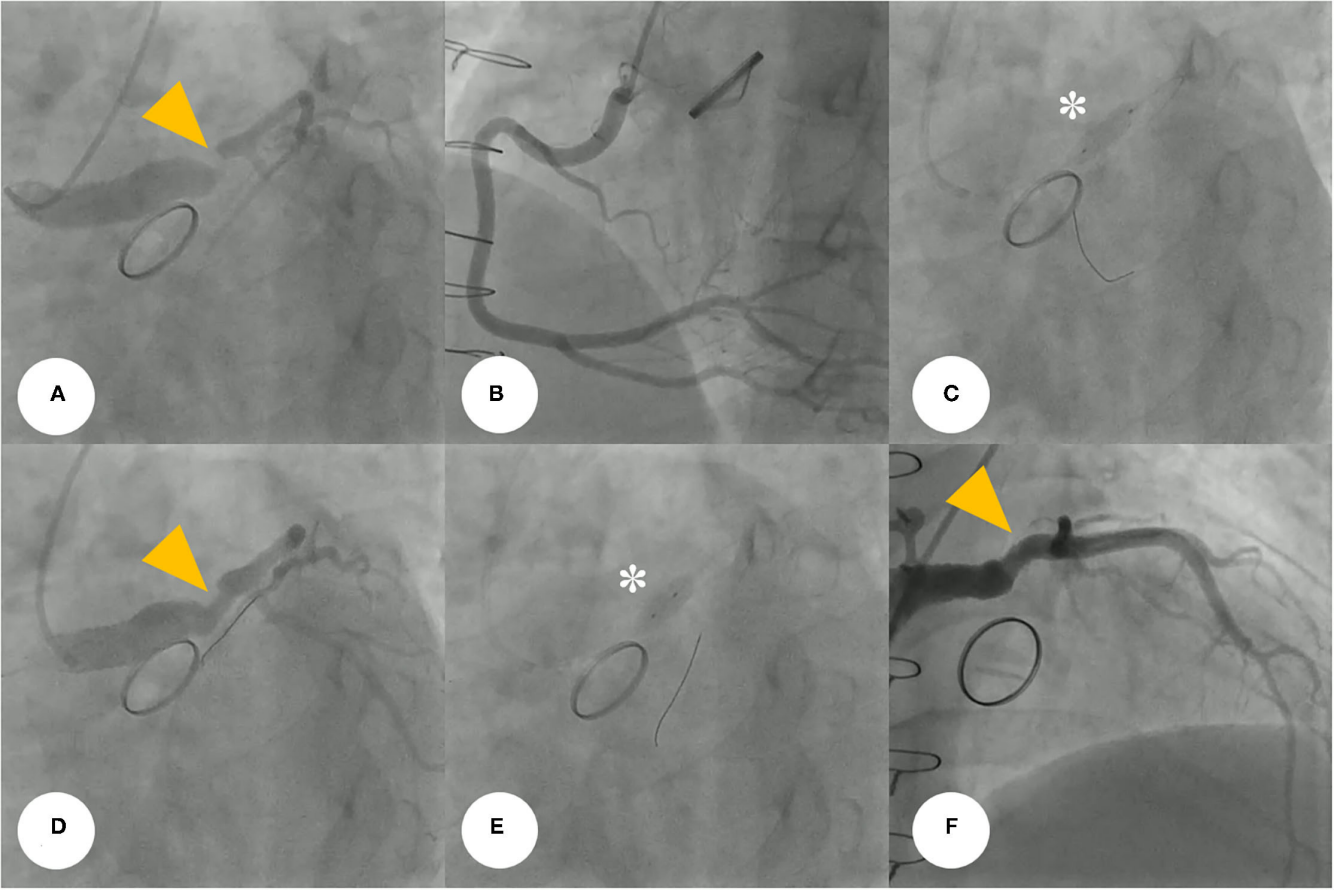
**(A)** Coronary angiogram demonstrates severe post-anastomotic left main coronary artery stenosis (yellow arrowhead) in a patient with Behçet's disease after a previous modified Bentall procedure. **(B)** Right-selective coronary angiogram shows no significant stenosis. **(C)** For this stenotic lesion, we used a drug-eluting stent (asterisk) (4.5 × 12 mm, Synergy^TM^, Boston Scientific, Marlborough, MA, USA). **(D,E)** The repeated coronary angiogram shows residual stent under-expansion (yellow arrowhead), hence, a non-compliant balloon (asterisk) (5.5 × 8 mm, Raiden 3^TM^, Kaneka Corporation, Osaka, Japan) was post-dilated. **(F)** Final coronary angiogram showing an optimal angiographic result (yellow arrowhead).

**Figure 5:**
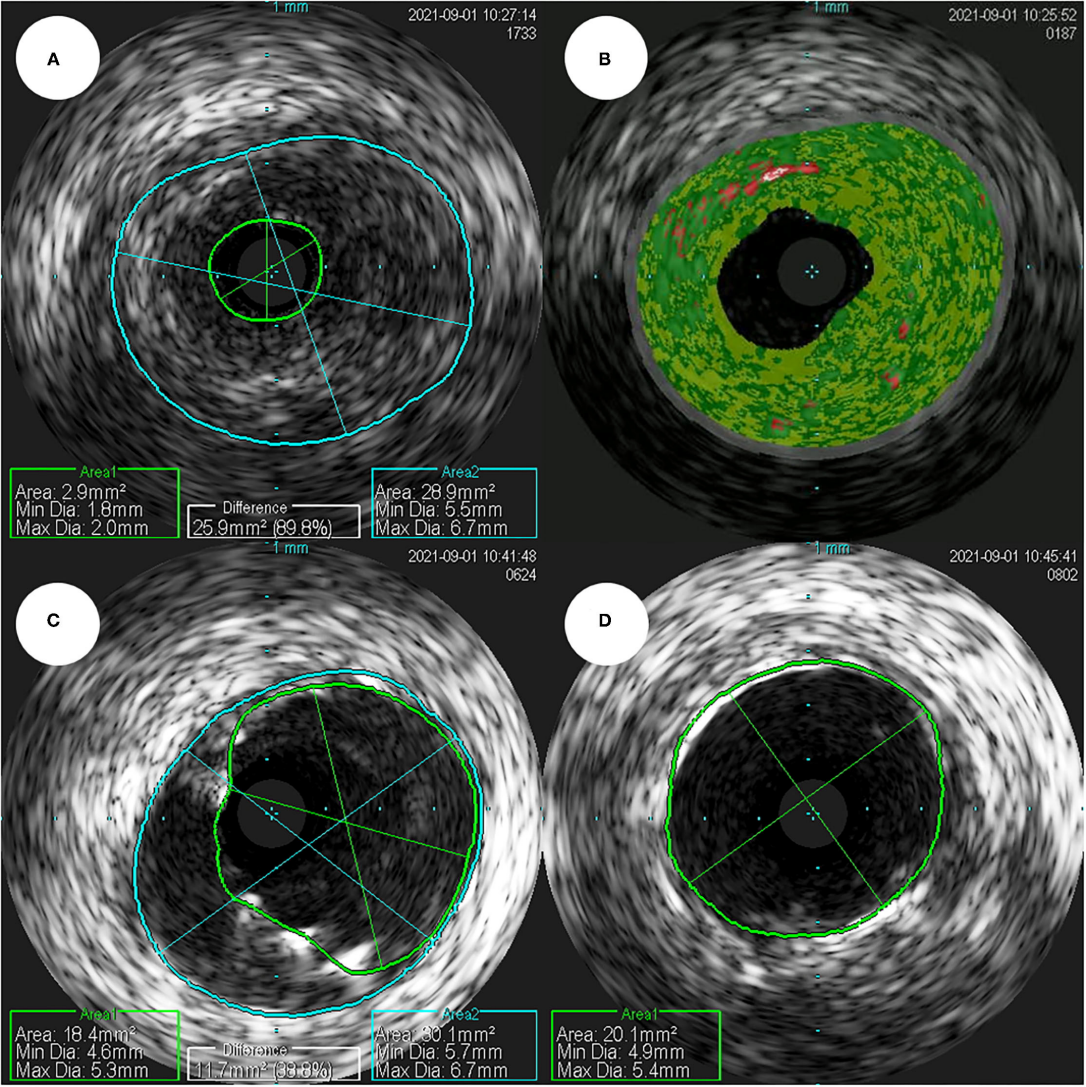
**(A)** In a pre-percutaneous coronary intervention intravascular ultrasound, the smallest lumen area as seen, was estimated to be 2.9 mm^2^ (plaque burden was estimated to be 89.8%). **(B)** In a virtual histology-intravascular ultrasound study, the fibrotic plaque shows mainly fibrous tissue, but no confluent necrotic core. **(C)** In a post-percutaneous coronary intervention intravascular ultrasound, the minimum stent area was estimated to be 18.4 mm^2^. **(D)** The repeated intravascular ultrasound shows optimized deployment of a drug-eluting stent with the full lesion coverage.

Coronary angiography (CAG) was performed through the right femoral artery during cardiac catheterization. Critical stenosis was observed at the anastomotic site between the LMCA and the conduit (Figure 4A; Supplementary Video 1). Right-sided CAG showed no significant stenosis, with collateral flow toward the left coronary artery (Figure 4B; Supplementary Video 2). After a 6F Judkins guiding catheter was engaged at the ostium of the left Cabrol-type composite graft, a 0.014-inch guidewire (Runthrough®, Terumo, Tokyo, Japan) was crossed through the LMCA to the left anterior descending coronary artery. Pre-PCI IVUS was performed using a guidance system (Eagle Eye® Platinum RX Digital IVUS Catheter, Volcano Corporation, Rancho Cordova, CA, USA), and a focally kinked and narrowed lesion with a minimum lumen area of 2.9 mm^2^ was observed (Figure 5A; Supplementary Video 3). In the VH-IVUS, fibrotic plaque was seen with mainly fibrous tissue but without a confluent necrotic core (Figure 5B; Supplementary Video 4). Thereafter, PCI was performed using a drug-eluting stent (4.5 × 12 mm, Synergy^TM^, Boston Scientific, Marlborough, MA, USA) (Figure 4C; Supplementary Videos 5 and 6). Immediately after the stent deployment, IVUS demonstrated an under-expanded stent strut with a minimum stent area of 18.4 mm^2^ (Figure 5C). Repeated CAG showed improved stenosis but under-expanded stent strut (Figure 4D; Supplementary Video 7). For this reason, the lesion was post-dilated using a noncompliant balloon (5.5 × 8 mm, Raiden 3^TM^, Kaneka Corporation, Osaka, Japan) to mitigate stent under-expansion (Figure 4E; Supplementary Video 8). A repeat IVUS revealed well-apposed and optimized deployment of the drug-eluting stent with full lesion coverage (Figure 5D; Supplementary Video 9). The final CAG also showed optimal angiographic results (Figure 4F; Supplementary Video 10).

After the PCI procedure, the patient received optimal medical therapy, including dual antiplatelet agents–aspirin (100 mg/day) and clopidogrel (75 mg/day), an oral anticoagulant–warfarin (1 mg/day), a high-intensity statin–rosuvastatin (20 mg/day), a beta-blocker–bisoprolol (2.5 mg/day), and an angiotensin II receptor blocker–valsartan (40 mg/day). The post-PCI coronary computed tomography angiography demonstrated a well-expanded and well-apposed stent strut at the anastomotic site (Figure 3B). His anginal symptoms dramatically improved, and he was successfully discharged from our hospital.

## Discussion and Conclusion

Behçet's disease is a multi-systemic illness, mainly manifested as mucocutaneous lesions (oral ulceration, genital ulcer, and other skin lesions), joint symptoms and signs (arthritis and arthralgia), eye lesions, and systemic vasculitis (7, 8). In addition, it may involve a variety of organs including the cardiovascular system (9, 10). Although it is still unclear as to which factor contributed to the development of stenosis at an anastomotic site, we presume that this fibrotic change was due to an aggressive healing process after the surgery, and Behçet's disease particularly contributed to the acceleration of this inflammatory response. Although the patient had received anti-inflammatory agents with good adherence, there would have been existing recurrent inflammatory insults considering the fluctuating levels of inflammatory markers (erythrocyte sedimentation rate), as shown in Figure 1. In other words, repeated inflammatory response toward Behçet's disease would have resulted in the development of fibrous plaques while inhibiting the healing process of the anastomotic site. The present study describes a successful PCI procedure for focal stenosis at an anastomotic site in a patient with Behçet's disease who previously received an aortocoronary graft anastomosed to the LMCA. During the PCI procedure, we evaluated the stenotic characteristics, confirming this fibrous change using VH-IVUS.

In a PubMed search, we found 11 successful PCI cases of anastomotic stenosis or occlusion between the aortocoronary conduit and the LMCA (3–5, 11–18). These results are summarized in Supplementary Table 1. In these studies, all patients were male and had underlying aortic conditions; 9 and 6 patients were diagnosed with AMI and cardiogenic shock, respectively and 9 patients survived. Thus, most cases presented as AMI, as seen in our case, and all patients had severe impairment of myocardial performance, leading to cardiogenic shock. To date, the present study describes an IVUS-guided PCI to the anastomotic site between the graft and the LMCA after a modified Bentall procedure. To our best knowledge, the present study is the first to investigate the characteristics of this lesion through VH-IVUS, which demonstrated the presence of a high content of fibrous plaques at the anastomotic site. Although VH-IVUS is considered an outdated analytic technique, and there are better methods to evaluate the plaque composition, including iMAP^TM^ IVUS, near-infrared spectroscopy, and optical coherence tomography, it is true that VH-IVUS provides accurate information about this lesion compared to IVUS alone. In this present case, it is particularly interesting to confirm the fibrous change caused by the inflammatory change through VH-IVUS in our patient with Behçet's disease.

The patient in the present case underwent PCI for anastomotic stenosis, instead of quadruple open-heart surgery owing to increased surgical risk associated with the latter. Despite many cardiothoracic surgeons having recently favored coronary artery bypass grafting as the treatment of choice (15), using autologous arterial grafts to artificial or vein grafts, it seems that a non-surgical intervention would have been a better treatment option in terms of patient safety. Furthermore, even after implantation of this stent, stenosis/occlusion at this anastomotic site could recur through the same inflammatory reaction. Although most published studies describe PCI or surgery as appropriate treatment strategies for the management of primary stenosis, little is known about an appropriate prevention strategy for its recurrence. However, regarding this case, it is hypothesized that both intensive medical treatment and active surveillance are needed to suppress the inflammatory reaction that can aggravate due to underlying Behçet's disease.

In conclusion, the IVUS radiofrequency data allow for a detailed assessment of plaque composition *in vivo*, therefore, the present case will provide new insights into the histopathological nature of stenotic lesions at the anastomotic site, especially in patients with chronic inflammatory diseases like Behçet's disease.

## Data Availability Statement

The original contributions presented in the study are included in the article/Supplementary Material, further inquiries can be directed to the corresponding author/s.

## Ethics Statement

The studies involving human participants were reviewed and approved by the Institutional Review Board of Chonnam National University Hospital (IRB No. CNUH-EXP-2022-027). Written informed consent for participation was not required for this study in accordance with the national legislation and the institutional requirements.

## Author Contributions

SO and JK drafted the manuscript. SO, DH, DS, and JK designed the study methodology. SO, DH, KC, DS, and JK collected the data. KC, MK, DS, YH, JK, YA, and MJ reviewed and edited the manuscript. All authors read and approved the final manuscript.

## Funding

This study was supported by a Grant (BCRI21074) of Chonnam National University Hospital Biomedical Research Institute.

## Conflict of Interest

The authors declare that the research was conducted in the absence of any commercial or financial relationships that could be construed as a potential conflict of interest.

## Publisher's Note

All claims expressed in this article are solely those of the authors and do not necessarily represent those of their affiliated organizations, or those of the publisher, the editors and the reviewers. Any product that may be evaluated in this article, or claim that may be made by its manufacturer, is not guaranteed or endorsed by the publisher.

## Supplementary Material

Supplementary Figure 1 The electrocardiogram also revealed ST-segment elevation in aVR and V1, with ST-segment depression in lead I, II, and aVL and precordial leads V4–6, suggesting LMCA occlusion. LMCA, left main coronary artery.

Supplementary Figure 2 Chest radiography showed mild cardiomegaly and definite evidence of prior median sternotomy and valvular replacement.

Supplementary Table 1 Clinical characteristics of cases about successful PCI for aortocoronary graft-LMCA anastomosis in the literature review.

## Abbreviations

AMI: acute myocardial infarction
AoV: aortic valve
CAG: coronary angiography
IVUS: intravascular ultrasound
LMCA: left main coronary artery
PCI: percutaneous coronary intervention
VH-IVUS: virtual histology-intravascular ultrasound.
