# Supplementary figures and images for: Case Report: Intravascular Ultrasound-guided Intervention for Anastomosis Stenosis of the Left Main Coronary Artery Post-Cabrol Technique
Source: Front Cardiovasc Med. 2022 Mar 2;9:778815. doi: 10.3389/fcvm.2022.778815 (PMC8926074; doi:10.3389/fcvm.2022.778815)

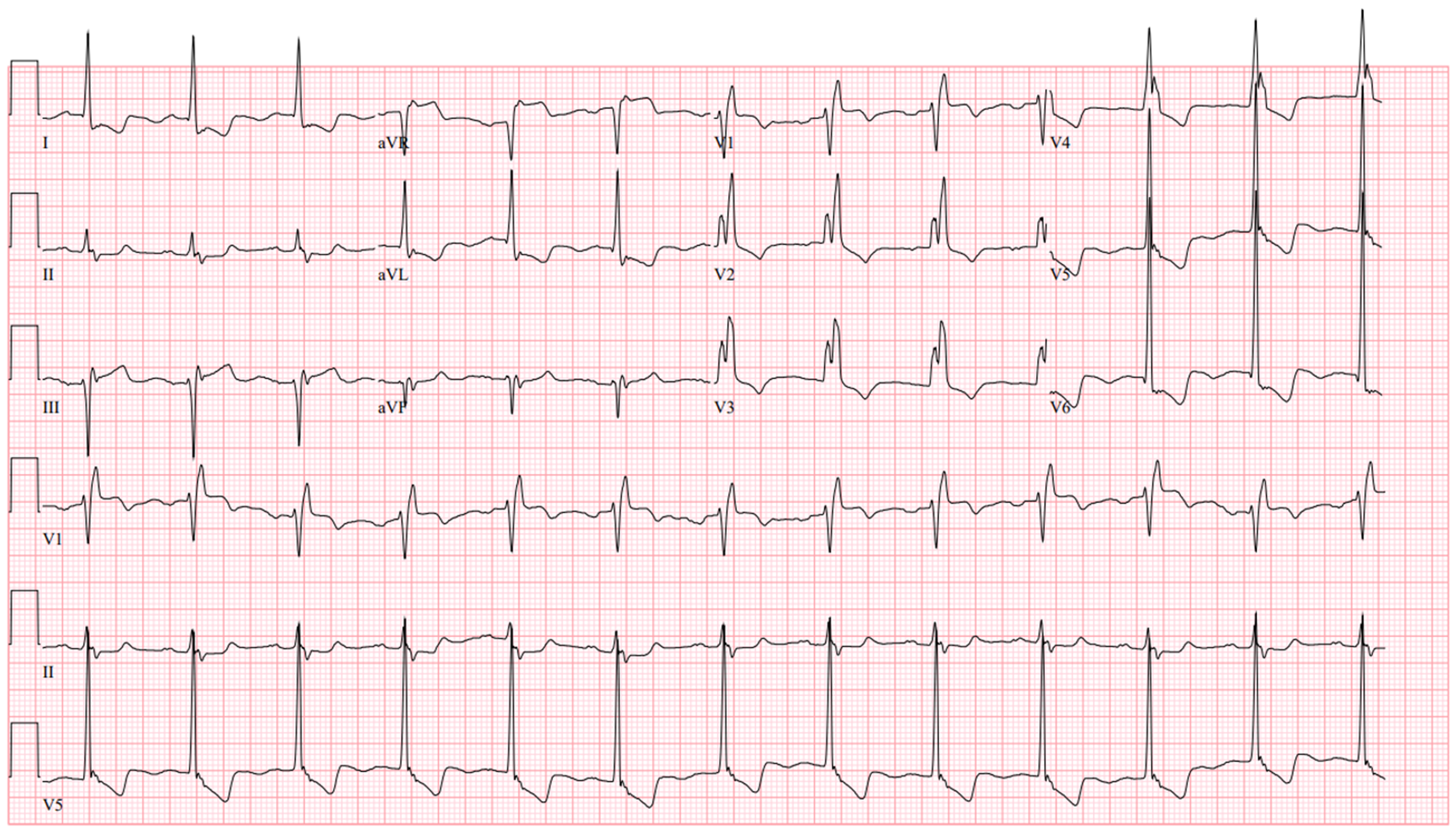

Supplement: Supplementary Figure 1 — The electrocardiogram also revealed ST-segment elevation in aVR and V1, with ST-segment depression in lead I, II, and aVL and precordial leads V4–6, suggesting LMCA occlusion. LMCA, left main coronary artery. [file Image_1.TIF]

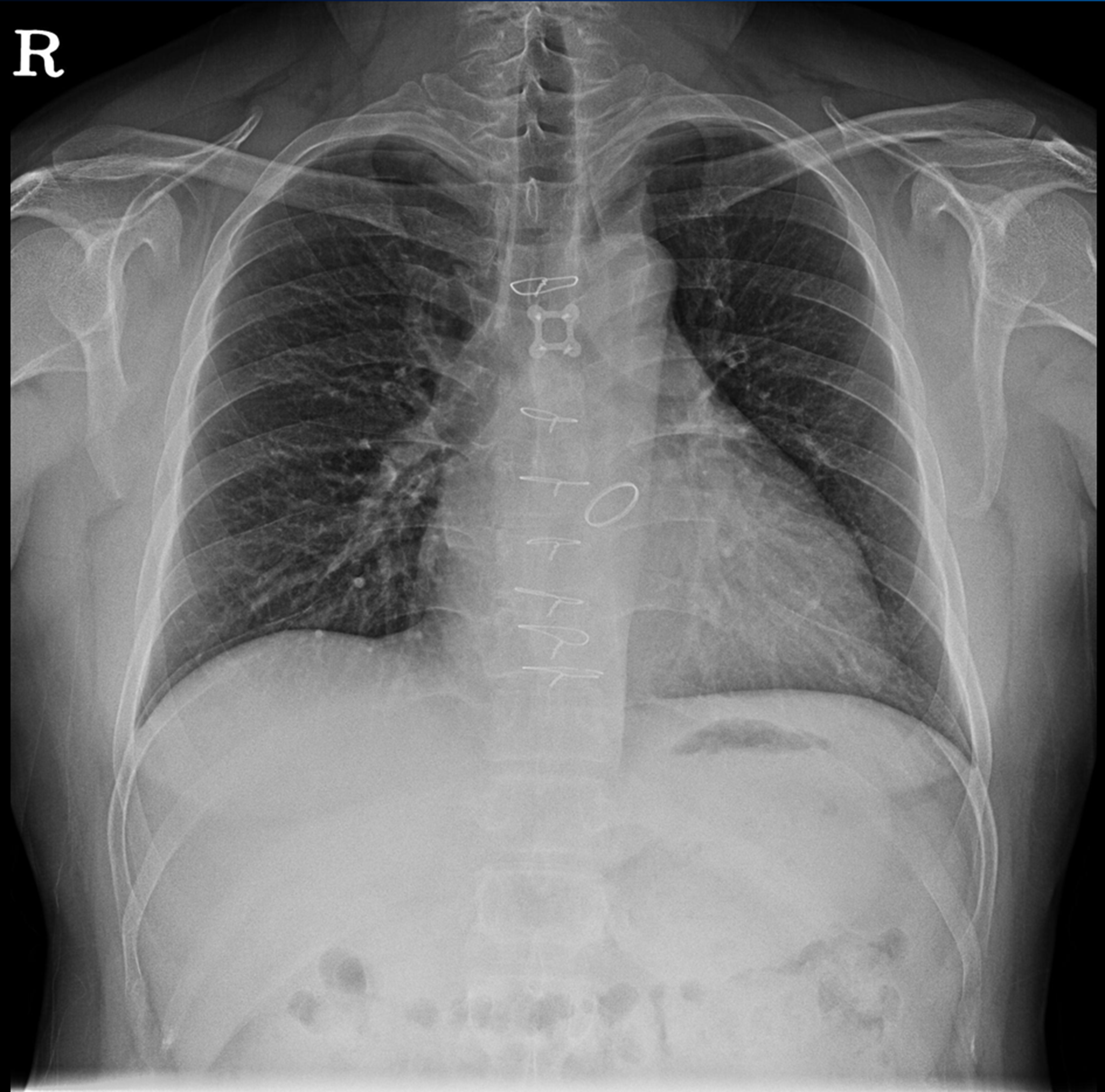

Supplement: Supplementary Figure 2 — Chest radiography showed mild cardiomegaly and definite evidence of prior median sternotomy and valvular replacement. [file Image_2.TIF]
