# Supplementary material for: Case Report: Intravascular Ultrasound-guided Intervention for Anastomosis Stenosis of the Left Main Coronary Artery Post-Cabrol Technique
Source: Front Cardiovasc Med. 2022 Mar 2;9:778815. doi: 10.3389/fcvm.2022.778815 (PMC8926074; doi:10.3389/fcvm.2022.778815)
Supplement: Supplementary Table 1 — Clinical characteristics of cases about successful PCI for aortocoronary graft-LMCA anastomosis in the literature review. [file Table_1.DOCX]

**Supplementary Table 1.** Clinical characteristics of cases about successful PCI for aortocoronary graft-LMCA anastomosis in the literature review.

| Authors | Nation | Year | Number of cases | Sex | Age (years) | AMI | CS | Diagnostic modalities | Vascular access | Underlying conditions | Survived |
| --- | --- | --- | --- | --- | --- | --- | --- | --- | --- | --- | --- |
| Coram, et al. | United States | 2005 | 1 | Male | 58 | 1 | 0 | CAG | Not mentioned | Aortic dissection | Yes |
| Wells, et al. | Austrailia | 2006 | 1 | Male | 49 | 1 | 1 | CAG | Not mentioned, maybe transfemoral | Aortic dissection | Yes |
| Hussain, et al. | Canada | 2006 | 1 | Male | 49 | 1 | 0 | CAG, CT | Not mentioned | Marfan syndrome, aortic aneurysm, aortic regurgitation | Yes |
| Bozlar, et al. | United States | 2008 | 1 | Male | 73 | 0 | 0 | CAG, CT | Not mentioned | Unknown | Yes |
| Hoskins, et al. | United States | 2010 | 1 | Male | 58 | 1 | 1 | CAG | Transfemoral | Aortic stenosis | No |
| Gonzalez, et al. | Mexico | 2012 | 1 | Male | 67 | 1 | 1 | CAG | Transradial | Aortic stenosis | Yes |
| Ikenaga, et al. | Japan | 2019 | 1 | Male | 17 | 1 | 1 | CAG | Transfemoral | Takayasu's arteritis | No |
| Komatsu, et al. | Japan | 2019 | 1 | Male | 64 | 1 | 0 | CAG, IVUS | Transfemoral | Aortic dissection | Yes |
| Fukuda | Japan | 2019 | 1 | Male | 44 | 1 | 1 | CAG, CT | Transfemoral | Aortic aneurysm | Yes |
| Siddiqui, et al. | United States | 2020 | 1 | Male | 61 | 1 | 1 | CAG | Transfemoral | Aortic stenosis | Yes |
| Karanfil, et al. | Turkey | 2020 | 1 | Male | 56 | 0 | 0 | CAG, CT | Transradial | Aortic aneurysm, mitral regurgitation | Yes |

AMI, acute myocardial infarction; CAG, coronary angiography; CS; cardiogenic shock; CT, computed tomography; IVUS, intravascular ultrasound; LMCA, left main coronary artery; PCI, percutaneous coronary intervention.
